# Immunohistochemical detection of Hsp90 and Ki-67 in pterygium

**DOI:** 10.1186/1746-1596-8-32

**Published:** 2013-02-21

**Authors:** Roberto Sebastiá, Marcelo Palis Ventura, Helena Parente Solari, Emilia Antecka, Maria Eugenia Orellana, Miguel N Burnier

**Affiliations:** 1 Department of Ophthalmology, Universitary Hospital Federal Fluminense University, Rio de Janeiro, Brazil; 2 Henry C. Witelson Ocular Pathology Laboratory, McGill University, Montreal, Canada

**Keywords:** Pterygium, Immunohistochemistry, Hsp90, Ki-67

## Abstract

**Background:**

To examine the immunohistochemical expression of heat shock protein 90 (Hsp90) and Ki-67 protein in human pterygium.

**Materials and methods:**

Tissues obtained during pterygium surgery of 15 patients who underwent the bare-sclera procedure and 10 normal conjunctivae were studied. All of these pterygia were primary ones. Recurrent pterygia were excluded. Normal bulbar conjunctivas (2 x 2 mm) were obtained from the nasal region close to the limbus from patients during their cataract and retina surgeries. Immunohistochemical detection of Hsp90 and Ki67 was done using the streptavidin–biotin method in paraffin embedded tissue sections.

**Results:**

The percentage of cells stained for Hsp90 was greater for pterygium epithelium (76 ± 10.8) than for normal conjunctiva (1.4 ± 0.8). In each pterigyum sample more than 60% of cells were positive. The differences in positive cells between normal and pterigyum epithelium were highly significant for Hsp90 (P < 0,001).

Pterygium epithelium also showed a higher percentage of cells that stained for Ki67 (10.1 ± 9.5) than for normal conjunctiva (2.1 ± 1.9). The differences in positive cells were also statistically significant for Ki67 (P < 0.01). Although there were significant differences in the majority of samples observed. It was noted that in some samples there was no difference between normal and pterygium epithelium for Ki67.

**Conclusion:**

Our results indicate an abnormal expression of Hsp90 and ki-67 in pterygium samples when compared to normal conjunctiva.The finding of abnormal expression of levels of Hsp90 in pterygium samples can stimulate new research into pterygium and its recurrence.

**Virtual Slides:**

The virtual slide(s) for this article can be found here: http://www.diagnosticpathology.diagnomx.eu/vs/1128478792898812

## Background

The pterygium is an ocular surface disease characterized by the centripetal growth of fibrovascular tissue associated with inflammation and vascularization. It can reach and invade the corneal surface reducing visual acuity [1]. Lacrimal distribution may be altered by the irregularities of the ocular surface causing keratoconjunctivities [2].

Epidemiological studies have shown that pterygium is strongly related to sun exposure, with little evidence that exposure during any particular period of life is more important than in other periods. The implication for prevention of pterygium is that ocular protection is beneficial at all ages, as this disease is linked to excessive ultraviolet (UV) radiation [3].

Although the major cause of pterygia has been attributed to UV-B radiation [4] many others etiological theories have been suggested [5] such as: cell cycle regulation [6,7], inflammatory mediators [8,9], immunological mechanisms [10], growth factors [11,12], angiogenic stimulation [13], extracellular matrix modulation [14,15], cholesterol metabolism modification [16], viruses [17] and hereditary factors [18].

At the moment, the treatment of pterygium is eminently surgical. There is a great recidive incidence using conventional techniques [2]. Some authors have reported the recurrence in about 50% of cases after excision [19]. A lower recurrence of pterygium is observed using conjunctival [20] or oral mucosa autograft [21,22]. The use of mitomicin drops reduces the incidence of recurrence of pterygium [23]. Since there is a high incidence of failure in the treatment of pterygium, recent studies have aimed at a better understanding of the pathophysiology of the disease in order to improve the therapy.

Although the pathogenesis of pterygium is yet undetermined, many features that suggest excessive or disordered growth have been found by different authors [7,24-26]. Tumorlike histologic characteristics, ranging from mild dysplasia to carcinoma in situ and local invasiveness, have been described [24].

The Ki-67 protein is well characterized on the molecular level and extensively used as a proliferation marker [27] and HSPs in the epithelia may be important in the surveillance of epithelial cell integrity and may represent a first line of defense against the transformation of epithelial cells induced by stress agents [28].

The aim of this study was to examine the expression of Hsp90 and Ki67 and correlate their presence with the pterygium etiology.

## Methods

Pterygium tissues obtained during pterygium surgery of 15 patients who underwent the bare-sclera procedure were included in this study. All of these pterygia were primary ones. Recurrent pterygia were excluded. Patients with previous ocular surgeries or with inflammatory or infectious disease were also excluded. None of these patients had an ophthalmic or systemic disease or used topical or systemic medication. Nine were men and six were women of ages that varied between 25 and 52 years (mean age 43 years). Except for local anesthetic no medicine or other chemical agent was used during pterygium excision. All cases were treated by the same surgeon (HPS) at the Universitary Hospital Universidade Federal Fluminense.

Normal bulbar conjunctivas (2 × 2 mm) were obtained from the nasal region close to the limbus from patients during their cataract and retina surgeries.

The tissues were then fixed using 10%paraformaldehyde and set in paraffin wax.

Informed consent was obtained according to the Declaration of Helsinki. Local (CEP-UFF) and national (CONEP) ethical committees had approved this study.

### Immunohistochemistry

The material was sent to the Department of Ophthalmology and Pathology at the McGill University Health Center and Henry C.Witelson Ocular Pathology Laboratory, Montreal, Quebec, Canada.

Formalin-fixed, paraffin-embedded sections of the specimens were H&E stained for histopathologic assessment by three ocular pathologists. Immunohistochemistry was done using the Ventana benchmark machine according to the protocol (Ventana Medical Systems, Inc.). The fully automated processing of bar code–labeled slides included baking of the slides, solvent-free deparaffinization, and CC1 [Tris-EDTA buffer (pH 8.0)] antigen retrieval. Slides were incubated with the monoclonal mouse anti-Hsp90 (StressGen, Victoria, BC, Canada) at a dilution of 1:50, others slides were incubated with the monoclonal mouse anti-Ki67 (Abcam, Cambrige, MA, USA) at a dilution of 1:50 for30 min at 37°C followed by application of biotinylated secondary antibody (8 min, 37°C) and then an avidin/streptavidin enzyme conjugate complex (8 min, 37°C). Finally, the antibody was detected in the presence of alkaline phosphatase enzyme by Liquid Fast-Red Substrate Kit (Abcam, Cambrige, MA, USA) and counterstained with hematoxylin. In the presence of alkaline phosphatase enzyme, Liquid Fast-Red produces a red reaction product that can be seen using microscopy. As a positive control, sections of colon cancer for Hsp90 and tonsil for Ki67 were used, and for negative controls, the primary antibody was omitted. The Hsp90 mainly exhibit citoplasmic immunoreactivity and ki67 immunostainig involves the nuclei of epithelial cells. The final reaction product has a reddish or pinkish color, varying in size and localization.

### Immunohistochemical evaluation

The evaluation of immunostaining expression for Hsp90 and Ki67 was calculated as a percentage of positive epithelial cells in relation to the total number in representative fields of pterigia and normal conjunctiva by the same three ocular pathologists.

### Statistical analysis

The unpaired Student’s t test was used to compare the samples. To determine the degrees of freedom the Aspin-Welch test was used. A P value of <0.05 was considered statistically significant.

## Results

**Figure 1:**
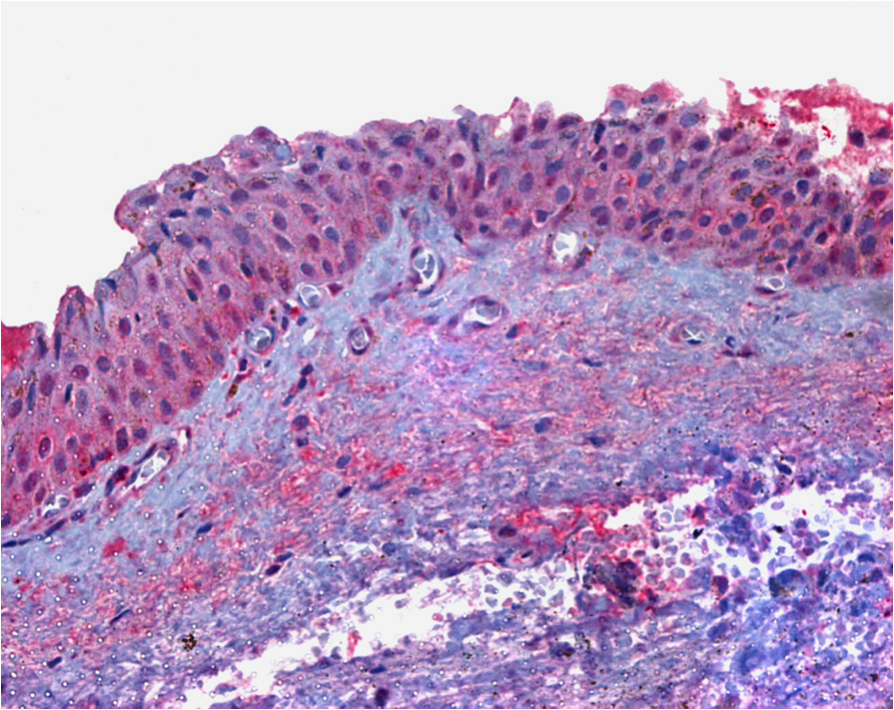
**Hsp90 immunostaining of pterygium tissue.** Strong positive cytoplasmic immunohistochemical reaction for Hsp90 protein in numerous epithelial cells of pterygium tissue (original magnification, X 400).

Ten normal conjunctivas and fifteen pterygia samples were studied. The number of cells stained for Hsp90 was greater for pterygium epithelium (76 ± 10.8) than for normal conjunctiva epithelium (1.4 ± 0.8) (Table 1). In each pterygium sample more than 60% of cells were positive (Table 2) (Figure 1). The differences in positive cells between normal and pterygyum epithelium were highly significant for Hsp90 (P< 0,001).

**Table 1:** Immunohistochemical staining of epithelial cells for Hsp90 and Ki67 in pterygium and normal epithelium

	**Hsp90 (% positive cells)**	**Ki67 (% positive cells)**
Normal conjunctiva	1.4	2.1
Pterygium	76*	10.1**

* P < 0,001 (versus pterigia and normal conjunctiva for Hsp90).

** P < 0,01 (versus pterigia and normal conjunctiva for Ki67).

**Table 2:** Immunohistochemical staining of epithelial cells for Hsp90 and Ki67 in pterygium and normal epithelia

	**Hsp90 % positive cells**	**Ki67 % positive cells**
Normal conjunctiva		
1	1	0
2	2	5
3	0	0
4	2	2
5	3	0
6	1	2
7	1	0
8	1	5
9	1	2
10	2	0
	Mean: 1.4 (SD 0.8)	Mean: 2.1 (SD 1.91)
Pterygium		
1	80	10
2	80	0
3	70	10
4	60	0
5	80	10
6	60	15
7	60	10
8	80	5
9	80	30
10	80	2
11	90	30
12	90	20
13	80	5
14	60	5
15	90	0
	Mean: 76 (SD 10.83)	Mean: 10.13 (SD 9.54)

**Figure 2:**
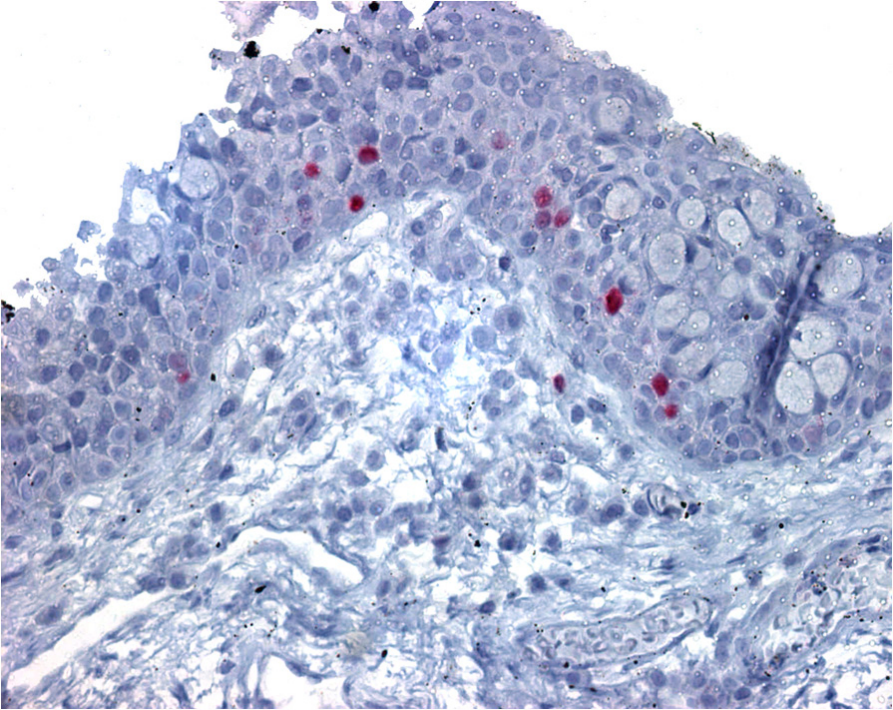
**Ki67 immunostaining of pterygium tissue.** Positive cell nuclei immunohistochemical reaction for Ki67 protein in relatively small number of epithelial cells of pterygium tissue (original magnification, X 400).

Pterygium epithelium also showed in a higher percentage of cells stained for Ki67(10.1 ± 9.5) than for normal conjunctiva (2.1 ± 1.9) (Table 1) (Figure 2). The differences in positive cells were also statistically significant for Ki67 (P < 0.01). Although there were significant differences in the majority of samples it was observed that in some samples there was no statistical significance between normal and pterygium epithelium for Ki67 (Table 2).

## Discussion

Pterygium is a chronic condition, characterized by the invasion of an altered ocular surface tissue, into the normal cornea [1,29].

Histologically, actively growing pterygium exhibit both degenerative and hyperplastic changes as well as proliferative and inflammatory disorders. They consist of an overlying conjunctival epithelium, which can appear normal or mildly hyperplastic. The underlying fibrovascular tissue usually presents a chronic inflammatory cellular infiltration and rich vasculature [30].

The pathogenesis of pterygium is still controversial. Many causative environmental factors may induce ocular inflammation, and various types of cytokines may cause abnormal cell changes of the ocular surface. UV-B exposure causes oxidative stress, leading to an excess of many potential mediators of pterygium growth [29]. The noxious effects of UV irradiation are caused either directly by UV phototoxic effects or indirectly by formation of radical oxygen species (ROS). ROS are very harmful to cells, because they injury cellular DNA, proteins, and lipids, called oxidative stress. ROS produced by UVB and UVA radiation through photosensitized oxidation can target DNA base guanine, giving rise to 8-hydroxydeoxyguanosine (8-OHdG) in DNA molecules. 8-OHdG has been shown to be a sensitive and stable biomarker for evaluating the degree of oxidative DNA damage [4].

Although pterygium is considered an oxidative-degenerative disease many authors believe that pterygium is a cellular proliferative disease probably induced by UV radiation. Growth factors have been studied for their role in pterygium development. They have been shown to be elevated in pterygium, playing an important role in cell proliferation, inflammation, connective tissue remodelling and angiogenesis [9,11,12,25,28,31,32].

A study of growth factors in cultured pterygium shows that active primary and recurrent pterygium fibroblasts immunoreact strongly with platelet-derived growth factor (PDGF) and basic fibroblast growth factor (b-FGF), respectively [9].

Incubation of pterygium-derived epithelium cells (PECs) and pterygium fibroblasts (PFs) with HB-EGF resulted in a significant increase in [3H] thymidine incorporation compared with that of control cells. HB-EGF stimulated chemotaxis of both PECs and PFs. These effects were abolished by the addition of a neutralizing antibody to HB-EGF [11]. HB-EGF also induces cell migration of both pterygium epithelium and fibroblasts. Previous studies found that b-FGF, VEGF and HB-EGF are upregulated after UV-B exposure [32].

The mRNA levels were evaluated for 5 peptide growth factors: transforming growth factor (TGFB1), vascular endothelial growth factor (VEGFA), basic fibroblast growth factor (FGF2), epidermal growth factor (EGF) and insulin-like growth factor (IGF1) in pterygium and phenotypically normal conjunctiva by employing qRTPCR which detected significantly higher levels of expression of FGF2 and VEGFA in the former, compared with the latter. Furthermore, FGF2 and VEGFA expression was significantly higher in more advanced pterygium, compared with less advanced ones, whereas VEGFA was also significantly correlated with postoperative recurrence. Results support the involvement of FGF2 and VEGFA in pterygium pathogenesis and imply that pharmacological modification of the clinical behavior of pterygium may be possible with agents directed against specific growth factors, such as VEGFA [32].

The cell cycle, specifically defective regulation of apoptosis, possibly plays a significant role in pterygium formation and growth [6,7]. The p53 tumor suppressor has been extensively investigated for its role in pterygium development. Increased p53 expression in the limbal epithelia of pingueculae, pterygium, and limbal tumors indicates the probable existence of p53 mutations in these cells at an early stage in their development, which is consistent with UV irradiation causation [6]. p53 acts as a checkpoint by preventing proliferation and subsequently inducing apoptosis if DNA mutations exist.

Disorganization in the extracellular matrix modulation has also been attributed to the pterygium formation [14,15].Pterygium epithelial cells (PEC) and limbal epithelial cells (LEC), but not normal conjunctival epithelial cells (CEC), respond to UVB exposure by an enhanced production of metalloproteinase-1 (MMP-1), an enzyme involved in the turnover of extracellular matrix, supporting the concept of UV-induced genetic trauma to LEC as a pathogenetic mechanism for pterygium [14].

### Hsp90

Hsp90 (heat shock protein 90) is a molecular chaperone and as its name implies, heat shock proteins protect cells when stressed by elevated temperatures [33,34]. It is an abundant and highly conserved molecular chaperone that is essential for viability in eukaryotes [35].

It has been estimated that Hsp90 accounts for 1% of the total soluble cytosolic protein in unstressed cells, making it one of the most abundant proteins [36].

Hsp90 promotes protein folding by preventing unfolded proteins from aggregating [37]. Similarly, it can prevent unfolding and aggregation of folded proteins, with which it is more or less stably associated. The first hints that Hsp90 may help stabilize metastable protein domains came from studies on steroid receptor complexes in the70s and 80s, and even preceded the formal identification of the 90-kDa component as Hsp90 [38].

It also stabilizes a number of proteins involved in tumor growth. Cancerous cells overexpress a number of proteins, including growth factor receptors, such as EGFR. Disregulation of these growth factor receptor pathways by over-expression or constitutive activation can promote tumor processes including angiogenesis and metastasis and is associated with poor prognosis in many human malignancies [39].

It was demonstrated that 17-allylamino-17-demetho-xygeldanamycin (17-AAG), the Hsp90 inhibitor can induce the degradation of mutant EGFR.

Inhibition of Hsp90 may induce apoptosis through inhibition of the PI3K/AKT signaling pathway and growth factor signaling generally [40].

The heat shock proteins (HSPs) induced by cell stress are expressed at high levels in a wide range of tumors and are closely associated with a poor prognosis and resistance to therapy. The increased transcription of HSPs in tumor cells is due to loss of p53 function and to higher expression of the proto-oncogenes *HER2* and c-*Myc*, and is crucial to tumorigenesis. The HSP family members play overlapping, essential roles in tumor growth both by promoting autonomous cell proliferation and by inhibiting death pathways. The HSPs have thus become targets for rational anti-cancer drug design: HSP90 inhibitors are currently showing much promise in clinical trials, whereas the increased expression of HSPs in tumors is forming the basis of chaperone-based immunotherapy [41].

Hsp90 is also required for induction of vascular endothelial growth factor (VEGF) and endothelial nitric oxide synthase (eNOS).Protein-protein interactions with the molecular chaperone hsp90 and phosphorylation on serine 1179 by the protein kinase Akt leads to activation of endothelial nitric oxide synthase. It was demonstrated that stimulation of endothelial cells with vascular endothelial growth factor recruits eNOS and Akt to an adjacent region on the same domain of hsp90, thereby facilitating eNOS phosphorylation and enzyme activation [35]. They are important for angiogenesis that is required for tumor growth.

In our study we found Hsp90 overexpressed in all pterygium samples (Mean: 76 ± 10.8). We observed statistical significance (p<0,001) if compared to normal conjunctiva (Mean: 1.4 ± 0.8). In some tumors, the same factors are involved in their etiopathogenesis [42,43] as well as in pterygium [9,11,12,14,15,26-30]. It was demonstrated that Hsp90 stabilizes many of these factors in tumors.

As far as we know this is the first report of Hsp90 expression in pterygium samples. Hsp90 is a protein involved in stress response and in normal homeostatic control mechanisms. Tumor cells require higher Hsp90 activity than normal cells to maintain their malignancy, so Hsp90 is a promising target for a pterygium treatment development [43]. We believe that searching for Hsp90 inhibitors (39, 40. 43) is of great importance and may represent a new therapeutic modality.

### Ki67

The expression of the human Ki-67 protein is strictly associated with cell proliferation. During interphase, the antigen can be exclusively detected within the nucleus, whereas in mitosis most of the protein is relocated to the surface of the chromosomes. The fact that the Ki-67 protein is present during all active phases of the cell cycle (G1, S, G2, and mitosis), but is absent from resting cells (G0), makes it an excellent marker for determining the so-called growth fraction of a given cell population [27].

It was demonstrated that despite the strong downregulation of pKi-67 expression in non-proliferating cells, the protein can nevertheless be detected at sites linked to ribosomal RNA (rRNA) synthesis [44]. Although this finding does not argue against the use of pKi-67 as a proliferation marker, it has wide ranging implications for the elucidation of pKi-67 function.

Inactivation of antigen KI-67 leads to inhibition of ribosomal RNA synthesis [46].

The usefulness of the Ki-67 labeling index has been well established for various types of malignant neoplasms [27]. In multivariate analysis, it was found that the Ki-67 labeling index is an independent and significant prognostic factor for disease-specific survival if all stage and grade categories are included [47].

In our study we found Ki67 overexpressed in 60% of pterygium samples (Mean: (10.1 ± 9.5). We observed statistical significance (p<0.01) if compared to normal conjunctiva (Mean: 2.1 ± 1.9). Although we observed negative results in 3 samples (30%) and 1 sample (10%) weakly stained. Garfias et al. [48] previously demonstrated expression of ki67 in epithelium of pterygium samples. The immunostaining was not observed in healthy conjunctivas. The results were in accordance with those found by Kase et al. [49]. It is interesting to note that we demonstrated great variance in Ki67 staining among the studied samples. This probably can indicate proliferative cell variability and should be related to recurrence rate. We also observed some samples of normal conjunctiva that showed positive staining and it can indicate that those samples could present some pathology not detected in biomicroscopy examination.

It was demonstrated that Ki67 is associated with cell proliferation.

Overexpression in malignant tumors means a great celular activity and poor disease prognostic [45,46]. Our finds proved the increase of cell proliferation in pterygium. The negative and weak results represent a minor activity and may be a better prognostic to recurrence of pterygium. We believe that new studies into the expression of Ki67 in pterygium are necessary for better understanding of the physiopathology of pterygium.

In this study abnormal Hsp90 and ki67 expression was found in pterygium epithelium, suggesting that the disease could be a result of uncontrolled cell proliferation. The staining pattern showed variations that could help understand the disease progress thus strategies in cell proliferation may be appropriate in the management of pterygium or its recurrence.

## Conclusion

Our results indicate an abnormal expression of Hsp and ki-67 in pterygium samples when compared to normal conjunctiva. The finding of abnormal expression of Hsp90 in pterygium samples can stimulate new research about pterygium and its recurrence.

## Competing interest

All the authors declare that there is no competing interest.

## Authors’ contributions

RS: contributions to conception and design, or acquisition of data, or analysis and interpretation of data. Drafting the manuscript. MPV: contributions to conception and design, analysis and interpretation of data, revising the intellectual content. HPS: contributions to conception and design, acquisition of data, or analysis and interpretation of data. Drafting the manuscript. EA: contributions to conception and design, analysis and interpretation of data. MEO: contributions to conception and design, analysis and interpretation of data. MNBJr: contributions to conception and design, analysis and interpretation of data. Final approval of the version to be published. All authors read and approved the final manuscript.
